# Transoral awake state neuromuscular electrical stimulation therapy for mild obstructive sleep apnea

**DOI:** 10.1007/s11325-022-02644-9

**Published:** 2022-05-27

**Authors:** Brandon Nokes, Peter M. Baptista, Paula Martínez Ruiz de Apodaca, Marina Carrasco-Llatas, Secundino Fernandez, Bhik Kotecha, Phui Yee Wong, Henry Zhang, Amro Hassaan, Atul Malhotra

**Affiliations:** 1 Division of Pulmonary, Critical Care, Sleep Medicine and Physiology, University of California, San Diego, 9500 Gilman Drive, La Jolla, CA 92121, USA; 2 VA San Diego Healthcare System, San Diego, CA, USA; 3 Otorhinolaryngology Clinical Consultant, Clinica Universidad de Navarra, Pamplona, Spain; 4 Médico Especialista en Otorrinolaringología Y Cirugía de Cabeza Y Cuello, Hospital Universitario Doctor Peset, Valencia, Spain; 5 Queens Hospital, Barking Havering and Redbridge University Hospitals NHS Trust, Rom Valley Way, Romford, UK

**Keywords:** Obstructive sleep apnea, Upper airway training, Transoral neuromuscular electrical stimulation

## Abstract

**Introduction:**

Obstructive sleep apnea (OSA) is a common disorder with major neurocognitive and cardiovascular sequelae. The treatment of symptomatic patients with mild OSA remains controversial given that adherence to positive airway pressure (PAP) has historically been suboptimal. With this notion in mind, we assessed a daily transoral neuromuscular electrical stimulation (NMES) device for individuals with mild OSA.

**Methods:**

The sample represents a subset of participants with a baseline AHI 5–14.9 events/hour, drawn from a parent study which also included participants with primary snoring. Outcome measures for the current study included changes in apnea-hypopnea index (AHI), Epworth Sleepiness Scale (ESS), Pittsburgh Sleep Quality Index (PSQI) and snoring levels before and after use of the NMES.

**Results:**

Among 65 participants (68% men) with median age of 49 years (range 24 to 79) and median BMI of 27.7 kg/m^2^ (range 20 to 34), the NMES device was used daily for 6 weeks. We observed a significant improvement in the AHI from 10.2 to 6.8 events/hour among all participants and from 10.4 to 5.0 events/h among responders. Statistically significant improvements in the ESS, PSQI, objectively measured snoring, and bed partner-reported snoring were observed. Adherence among all participants was 85%.

**Discussion:**

This NMES device has the benefit of being a treatment modality of daytime therapy which confers a high level of tolerability and patient acceptance. It alleviates the need for an in situ device during sleep and leads to improvements in OSA severity, snoring, and subjective sleep metrics, potentially crucial in mild OSA. Further studies are needed to define which individuals may benefit most from the device across the wider spectrum of OSA severity and assess long-term therapeutic outcomes.

**Trial registration:**

ClinicalTrials.gov Identifier: NCT03829956.

## Introduction

Obstructive sleep apnea (OSA) is a common disorder with major neurocognitive and cardiovascular sequelae. Recent estimates suggest that nearly a billion adults (aged 30 to 69 years) worldwide have OSA with the majority suffering from a mild disease [1]. The decision to treat mild sleep apnea presents a challenge in clinical practice [2]. Some patients with mild OSA have a substantial burden of symptoms, whereas others may be at risk of disease progression over time, in part, due to weight gain [3]. A growing body of evidence suggests that mild OSA is associated with an increased risk of stroke [4], hypertension, impaired quality of life, and a predisposition to early atherosclerosis [5]. Moreover, treatment of mild OSA can improve blood pressure suggesting that diagnosing and treating mild OSA is of clinical value [6].

A number of options exist for the management of mild OSA. PAP therapy (positive airway pressure) remains the primary modality but adherence is challenging particularly with mild disease [7]. Lifestyle changes such as weight loss, oral appliances, and upper airway surgery, are alternative options although their efficacy among those with mild OSA is highly variable. Thus, there is a general acknowledgement that new therapies are required. Although the pathogenesis of sleep apnea varies across patients, anatomical compromise can be overcome by increased activity in pharyngeal dilator muscles [8]. As airway protective mechanisms vary across patients, pharyngeal collapse occurs in those who are anatomically susceptible. In addition, there are data suggesting the occurrence of upper airway neuromyopathy in some patients with snoring and OSA [9]. Upper airway “re-education therapy” [10] and inspiratory muscle training [11, 12] provide additional means through which patients with OSA may experience clinical improvement. Given the advancement in defining specific physiological traits and OSA endotypes [13], it is very likely that there is a subset of patients that is most amenable to upper airway training.

Recent work has shown that training of the upper airway dilator muscles using daytime neuromuscular electrical stimulation (NMES) is feasible and may lead to improvements in OSA severity. The current study expands on the previous work [14, 15] to test the hypothesis that daytime upper airway muscle electrical stimulation would lead to clinical improvements during sleep in those with mild sleep apnea. The study by Baptista et al. showed a significant improvement in primary snoring, but did not look explicitly at individuals with mild OSA given the patient heterogeneity [16]. This paper is a secondary analysis of the subset of these individuals with mild OSA.

## Methods

### Recruitment of study sample

**Fig. 1:**
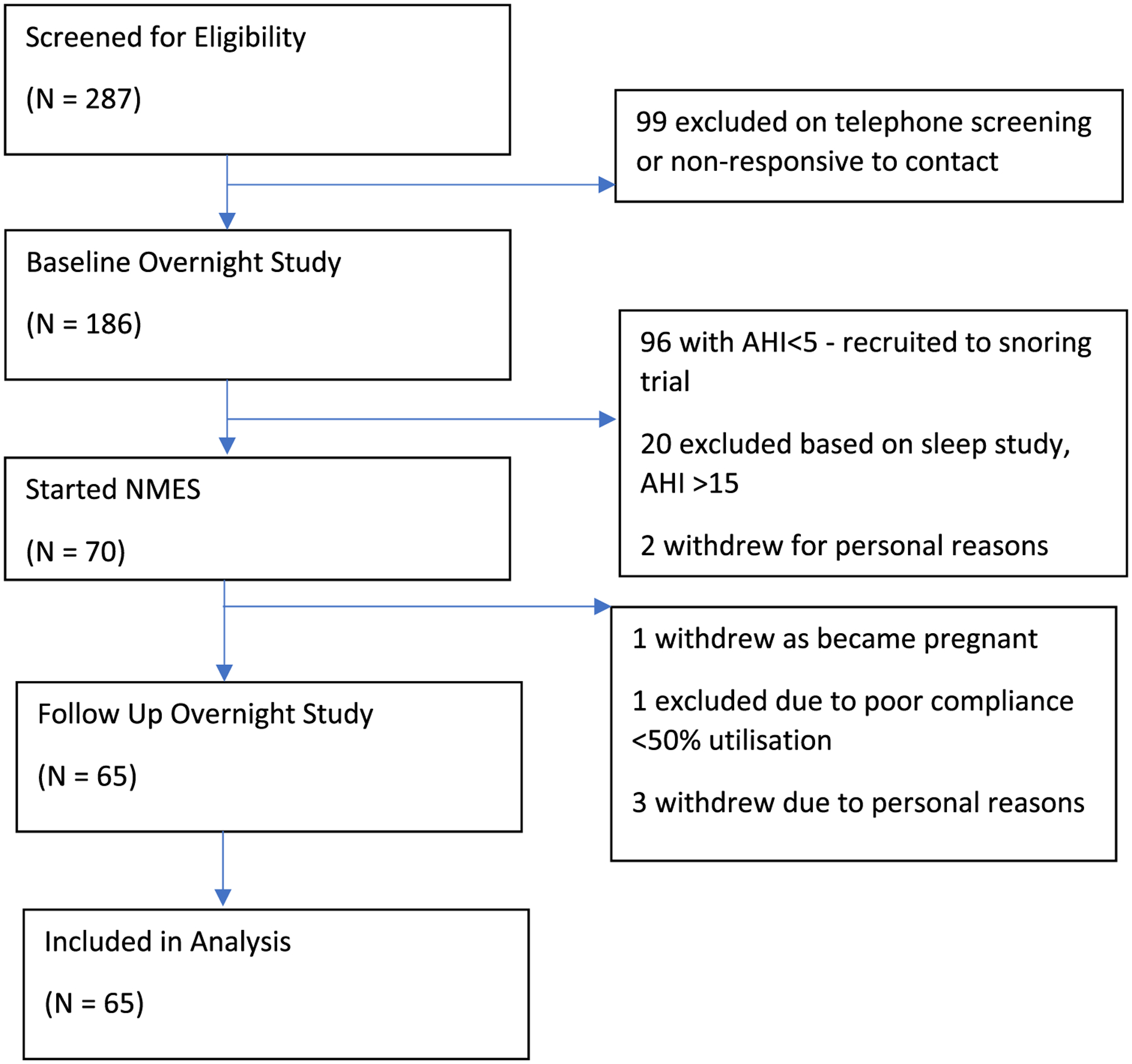
Trial enrollment flowchart. The flowchart delineates the number of patients who were screened and the various reasons for exclusion and dropout to arrive at our final sample size of 65

Patients referred to the Department of Ear, Nose, and Throat ambulatory clinic specializing in sleep-disordered breathing (SDB) were recruited from multiple centers including Queen’s Hospital, Romford (Barking, Havering and Redbridge NHS Trust), UK, Clinica Universidad de Navarra, Pamplona Spain, and Hospital Universitario Miguel Peset, Valencia, Spain. Inclusion criteria for the parent study included primary snoring and mild OSA, defined as an apnea–hypopnea index (AHI) < 15.0 events/h; the current analyses were restricted to those with an AHI ≥ 5 and < 15 events/h. Exclusion criteria included pregnancy, gross tonsillar hypertrophy, and placement of a pacemaker or electrodes. Following an initial telephone consultation, a two-night ambulatory sleep study was performed using the WatchPAT home sleep apnea test (Itamar Medical, Caesarea, Israel) [17], previously validated against polysomnography, followed by a clinical and endoscopic examination by an ENT surgeon, review of inclusion and exclusion criteria, and signing of informed consent (Fig. 1). Ethical approval for the study was sought and granted for this study by Stanmore Research Ethics Committee (REC) using the Integrated Research Application System (IRAS project ID: 219,271). In addition, approval by the local Ethical Committee of the Communities of Navarra and Valencia was obtained. The parent study was registered on clinicaltrials.gov (NCT03829956). The study was conducted in line with good clinical practice, ISO 14155:2011.

### Baseline assessments

For a period of 2 weeks prior to initiating NMES therapy, the bed partner completed a daily subjective assessment of snoring (visual analog scale: 1: no snoring through 10: intolerable snoring) and the average rating over the two weeks was used as the baseline. All study participants also completed the Pittsburgh Sleep Quality Index (PSQI) and Epworth Sleepiness Score (ESS) questionnaire. Following completion of the baseline assessments, participants were shown and instructed to use the NMES device once per day for the subsequent 6 weeks, which was the duration adopted in the previous proof-of-concept study [14].

### Transoral neuromuscular electrical stimulation

**Fig. 2:**
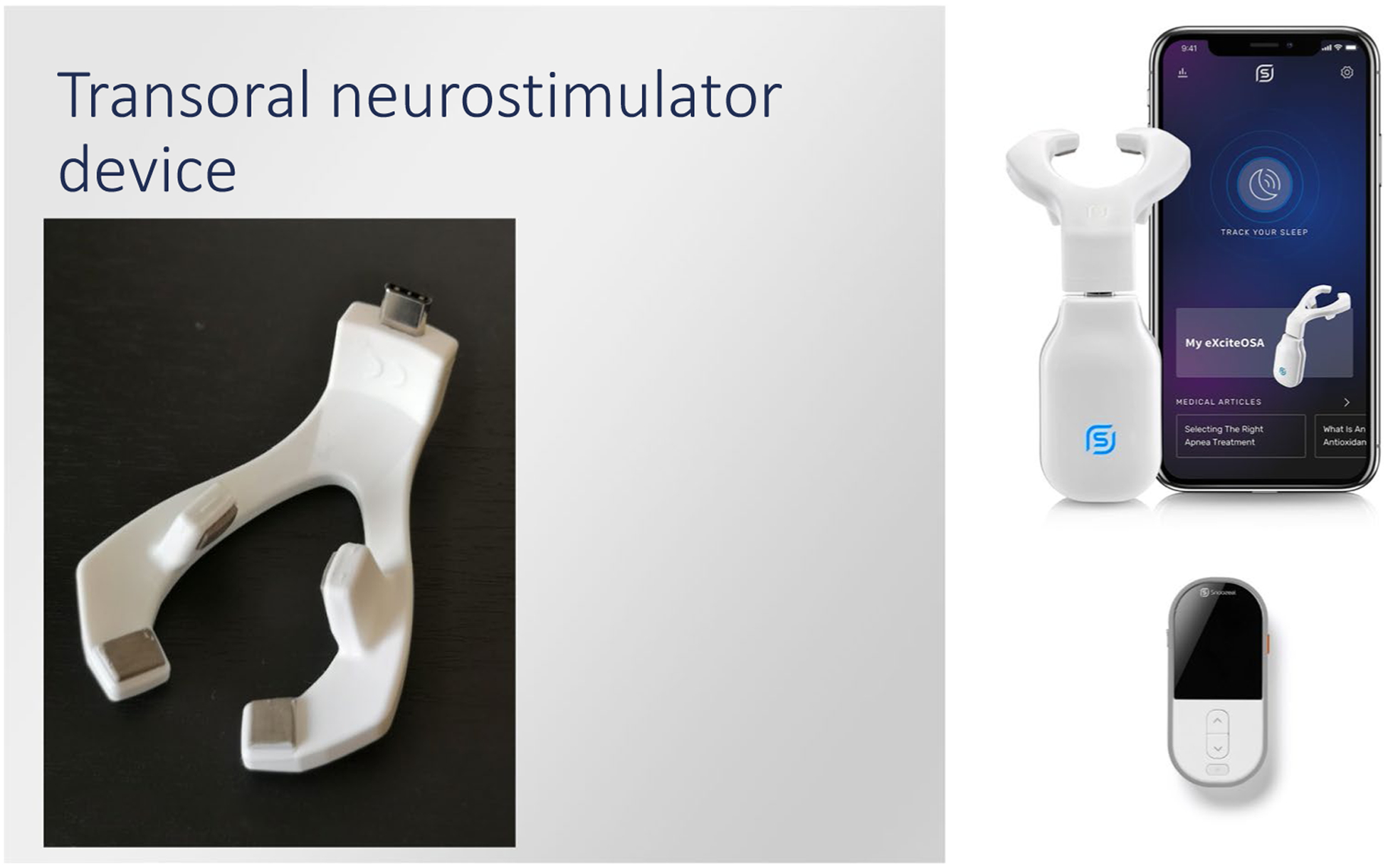
The exciteOSA device as well as associated application interface

A reusable eXciteOSA® device (Signifier Medical Technologies Ltd; London, UK), which functions by creating transoral neurostimulation of the tongue muscles via electrodes, was provided to each participant for 6 weeks. The device has three components: a control unit, a washable electrode mouthpiece, and a smartphone application. The device and associated application interface are shown in Fig. 2. The mouthpiece is connected to the control unit via a micro-USB port and a Bluetooth connection is established between the control unit and the smartphone app. This technology allows the user full control over the intensity levels during therapy sessions and on/off functionality of the control unit and mouthpiece. The mouthpiece is placed in the mouth, with two electrodes located above and two electrodes below the tongue. The therapy consists of a series of pulse bursts over a 20-min therapy period during which the pulse frequency changes in a defined sequence. The product was advised to be used at any time of day.

### Follow-up assessments

At the end of the intervention period, the two-night home sleep apnea test with the WatchPAT was repeated. As before, the average value of two recordings was used in the final analysis. Parameters from the WatchPAT included oxygen saturation, AHI, oxygen desaturation index (ODI) based on the 4% desaturation threshold, and percent time snoring. Hypopneas were scored based on the 2018 AASM scoring criteria for peripheral arterial tonometry [18]. WatchPAT reports were manually reviewed considering the limitations of auto-scoring in mild OSA. Subjective data on the ESS and PSQI were also acquired as were data on bed partner reports of snoring on the VAS. Finally, any side effects or adverse events were also recorded.

### Statistical considerations

To assess treatment effects, data from baseline and follow-up assessments were compared using a two-tailed paired *t*-test, with *p* ≤ 0.05 considered statistically significant. To examine whether factors such as age, sex, BMI, neck size, and clinical examination characteristics (Friedman Classification, tonsil size, Mullers maneuver, structural nasal obstruction) were associated with the pre-post change with therapy, multiple linear regression analyses were used. Given that specific demographic and clinical indicators associated with a response are not known, additional analyses were undertaken to characterize those with the greatest AHI decrease. A reduction in the AHI was taken as an indication of possible benefit and was considered the cutoff for being considered a responder [16].

## Results

Sixty-five participants (67.8% men) with mild OSA at baseline (average WatchPAT AHI ≥ 5 and < 15 events/h at baseline) completed the study with a median age of 49 years (range 24–79) and median BMI of 27.7 kg/m^2^ (range 20.4–33.8). Baseline participant and sleep characteristics are shown in Table 1.

### Impact of therapy on all participants with mild OSA

Following 6 weeks of therapy, there was a significant improvement in AHI, with a mean reduction of 3.4 ± 5.0 events/h (95% CI 2.2–4.7) from 10.2 to 6.8 events/h (*p* < 0.01). The ODI decreased by 2.5 ± 4.6 events/h (95% CI 1.4–3.6) from 8.4 to 5.9 events/h (*p* < 0.01). Time spent snoring > 40 dB decreased from 36.5 to 21.5% of the total WatchPAT recording time (reduction of 15.0 ± 15.4; 95% CI 11.1–18.8; *p* < 0.01). Mean ESS reduced from 8.7 to 5.3 (reduction of 3.4 ± 4.1; 95% CI 2.4–4.4; *p* < 0.01), and composite PSQI reduced from 7.3 to 5.9 (reduction of 1.4 ± 2.8; 95% CI 0.7–2.1; *p* < 0.01) (Table 2). There was a fairly consistent improvement with the use of the device for both objective and subjective parameters. Mean bed partner-reported snoring decreased from 6.3 to 3.9 (reduction of 2.4 ± 1.8; 95% CI 1.9–2.9; *p* < 0.01). The mean baseline oxygen saturation was 95% and did not significantly change over time.

### Responder analyses

Within our study, there was a consistent improvement in OSA, although with some associated variability. Within the study sample cohort, 51 out of 65 (78%) of participants experienced some reduction in their AHI and were considered “responders” (see Table 3). These participants exhibited a change in the mean AHI from 10.4 to 5.0 events/h with a mean change of 5.4 ± 2.8 events/h (95% CI 4.7–6.2; *p* ≤ 0.01). In this group, the ODI reduced from 8.6 to 4.3 events/h (mean reduction of 4.3 ± 2.7 events/h) and ESS from 9.3 to 5.4 (mean reduction of 3.9 ± 3.7; /24 points), supporting the change in AHI with other objective and subjective indices. Regression analyses of these responders determined that younger age (B − 0.05, SE 0.02, *p* = 0.04) and higher Friedman oral cavity score (B 0.94, SE 0.44, *p* = 0.03) were predictive of AHI reduction with device use. Other factors such as neck circumference, tonsil size, other demographic data, structural nasal restriction, and endoscopic examination findings were not associated with the degree of AHI reduction.

### Therapy adherence

Participant adherence (defined as the percentage of days during which a 20-min therapy session was completed) was 85% (range 57–100%). Adverse events were minor, infrequent, and transient including excess drooling, tongue tingling/discomfort, tooth discomfort, and gagging. No serious adverse events were reported.

## Discussion

The results of this study show that daytime transoral neuromuscular training for mild OSA was associated with improvements in disease severity and accompanying symptoms of snoring, sleepiness, and overall compromise in sleep quality. Our findings are important for a number of reasons. First, we have seen improvements in mild sleep apnea, such that the resulting average AHI falls within a normal range among responders. Second, we have observed improvements in both objective and subjective snoring using validated objective snoring measurements from sleep studies and bed partner diaries. Third, the NMES technology was well tolerated with no serious adverse events reported. Unlike PAP or oral appliances that require night-time use, the NMES device used in this study is a daytime therapy device with a low burden of use for the patient. This approach makes patient tolerability and the acceptance of the therapy much more feasible. Thus, we believe that this new technology is worthy of further study and consideration for the treatment of mild OSA.

A number of options exist for the treatment of mild OSA but many of these modalities are hampered by poor patient adherence and intolerance. PAP provides benefits for some patients and is widely considered to be the gold standard treatment of OSA. Historically, this therapy is variably tolerated by patients, with one study estimating long-term adherence with PAP to be anywhere from 40 to 85% [19].

Alternative therapies used to manage mild OSA include oral appliances, upper airway surgery, and diet/exercise. The efficacy of these treatments is highly variable and we lack a robust ability to predict who is likely to respond [20, 21]. Oral devices and appliances are not a homogenous group as they differ greatly in both design and action, which makes their effectiveness difficult to predict.

Hypoglossal nerve stimulation (HNS), a surgically implanted nerve stimulator to overcome obstructive events by tongue stimulation during sleep, has become commercially available in recent years. They are indicated for patients with moderate to severe OSA who have failed CPAP therapy. HNS devices have been associated with complications such as infections and device malfunction [22]. The cost of HNS remains a major limiting factor for widespread adoption and the cost-effectiveness of HNS devices continues to be uncertain [23].

While upper airway surgery remains another treatment choice, the surgical treatment for OSA in adults has traditionally been considered of variable benefit [21]. Sethkumar et al. noted that the wide range of surgical procedures available made site-specific and targeted surgery with rigorous and correct patient selection critical to achieve optimal results [24]. The stringent nature of the selection criteria limits the number of patients who can benefit from this treatment.

Training of the upper airway musculature and its relationship to improved OSA is not a new concept. A paper in the BMJ in 2006 showed that the use of the didgeridoo led to improvements in sleep-disordered breathing [25]. The use of this instrument does require considerable pharyngeal muscle activation and thus in theory the instrument could be training the dilator muscles of the upper airway yielding benefits during sleep. A group in Brazil has also reported that a defined upper airway muscle exercise regimen could improve sleep-disordered breathing among participants although the mechanisms behind this finding are unclear [26]. Other studies have found that corresponding oropharyngeal exercises can alleviate moderate OSA [27]. The former paper was a systematic review and meta-analysis where the authors stipulated that these positive effects were caused by a change in oropharyngeal muscle tone.

However, exercise-based approaches require practice/training and are probably implemented with considerable variability. While NMES does require daily practice, the ease of use and patient-titrated effect may allow for more sustained, longitudinal benefits. The principle of NMES has been attempted in the treatment of OSA. In a randomized, placebo-controlled study on electrical stimulation of the tongue musculature, Randerath et al. noted a significant effect on snoring although AHI remained unchanged [28]. This study included patients with moderate and severe OSA (baseline AHI 10–40 events/hour) and a device that used electrodes positioned in the submental area relying on transcutaneous stimulation through the neck externally. Our trial included patients with mild OSA only and is an entirely intraoral device with transmucosal stimulation directly onto the tongue muscles. Our results support clinically and statistically significant improvements in AHI, objective snoring sound as well as the subjective measures of daytime sleepiness (ESS), sleep quality (PSQI), and bed partner reported snoring (VAS).

Despite our study’s strengths, we acknowledge a number of limitations. First, the absence of a sham comparator means that the observed improvements may have been a result of a placebo effect and/or non-specific changes in health behavior such as diet, exercise, or alcohol intake. However, we did not instruct our participants on any of these factors and thus doubt any major change in health behavior during our study given the longstanding nature of the snoring complaints and short therapy period of 6 weeks. Furthermore, the possibility of a placebo effect is partially mitigated by the inclusion of objectively assessed endpoints, and the fact that consistent changes were observed for objectively measured and self-reported endpoints. However, ongoing clinical trials do include a control arm (https://clinicaltrials.gov/ct2/show/NCT05252156). Second, the open-label nature of the study prevented the sleep study staff from being blinded to the pre or post-therapy status of the patient. Importantly, however, the sleep studies were analyzed using the validated auto-scoring algorithms in the zzzPAT software (Itamar Medical), with manual adjustment limited to reviewing the signals for irregularities/artifacts, thus minimizing the possibility that the lack of blinding impacted the interpretation of the objective snoring, AHI, or ODI endpoints [29, 30]. Additionally, the primary statistical analysis was performed by an independent statistician. Third, we did not assess hard outcomes such as cardiovascular disease endpoints or neurocognitive performance. These outcomes remain the key endpoints to consider and should be assessed in larger long-term trials. Although we have demonstrated a reduction in clinical indices associated with sleep-disordered breathing, we cannot conclusively determine whether our interventions result in the desired improvements in OSA complications. Ongoing clinical trials include polysomnography in order to assess better the differences in rapid eye movement (REM) versus non-REM AHI as well as the impact of sleep position. Additionally, it bears mention that we excluded individuals with a BMI > 35 kg/m^2^ as we believe it is less likely that NMES will have a treatment effect in morbid obesity. Finally, we did not study the mechanisms underlying our observed improvements, but have ongoing efforts to record genioglossus muscle function before and after stimulation to explore these mechanistic aspects (NCT03913494). In addition, we believe that our therapeutic approach may be helpful for particular OSA endophenotypes (such as those with lower upper airway gain) which we plan to study in future trials [31].

Despite these limitations, we believe that our new findings are of interest and represent an early step in a research pathway worthy of further pursuit and consideration for therapeutic options for mild OSA. We considered this single-arm trial design to be an appropriate next step in order to define the impact of NMES over time before proceeding to subsequent trials incorporating a non-therapeutic comparator, followed by comparative-effectiveness trials. Future studies will be able to include the impact of time of day device use on sleep outcomes as well as provide more granular adherence/individual monitoring of device settings.

## Conclusion

Mild OSA continues to be largely unaddressed as the more severe variants of this disease take up a large amount of attention; however, the literature on mild OSA provides evidence that it can be symptomatic, lead to adverse sequelae, and that its early treatment may lead to the improvement of future health outcomes [13]. We have tested a robust approach that uses electrical stimulation of upper airway muscles during wakefulness. This device has the benefit of being a daytime sleep therapy appliance which confers a high level of tolerability and patient acceptance. It also leads to improvements in indices of mild OSA, objectively measured and bed partner-reported snoring, and subjective sleepiness and sleep quality indices, which may represent an important future step for treating these afflicted patients.

## Figures and Tables

**Table 1:** Baseline clinical characteristics

	N	Median	Lower quartile	Upper quartile	Range
Age (years)	65	49	35.5	57.0	24–79
BMI (kg/m2)	65	27.7	26.0	30.0	20.4–33.8
AHI (events/hour)	65	11.4	6.6	12.9	5.1–15.0
ESS (events/hour)	65	8	4.0	13.5	0–22
Alcohol (unit/wk)	61	2	0	10	0–40
Smoking (pack years)	61	0	0	0	0–30
Neck circumference (inches)	65	15.5	14.4	16.3	12.3–18.5

**Table 2:** Changes in sleep indices pre- to post-therapy among all participants

	Pre-therapy	Post-therapy	Change (mean ± SD)	95% CI	2-tailed *p*-value
AHI (events/hour)	10.2	6.8	3.4 ± 5.0	2.2–4.7	< 0.01
ODI 4% (events/hour)	8.4	5.9	2.5 ± 4.6	1.4–3.6	< 0.01
Objective snoring > 40 dB (%)	36.5	21.5	15.0 ± 15.4	11.1–18.8	< 0.01
ESS (/24 points)	8.7	5.3	3.4 ± 4.1	2.4–4.4	< 0.01
PSQI (21 points)	7.3	5.9	1.4 ± 2.8	0.7–2.1	< 0.01

**Table 3:** Changes in sleep indices pre-to post-therapy among responders

	Pre-therapy	Post-therapy	Change (mean ± SD)	95% CI	2-tailed *p*-value
Any reduction in AHI (n = 51)					
AHI (events/hour)	10.4	5.0	5.4 ± 2.8	4.7–6.2	< 0.01
ODI 4% (events/hour)	8.6	4.3	4.3 ± 2.7	3.6–5.1	< 0.01
Objective snoring > 40 dB (%)	37.1	20.2	16.9 ± 16.7	12.2–21.6	< 0.01
ESS (/24 points)	9.3	5.4	3.9 ± 3.7	2.8–4.9	< 0.01
PSQI (21 points)	7.2	5.5	1.7 ± 2.3	1.0–2.3	< 0.01
≥ 50% reduction in AHI (n = 28)					
AHI (events/hour)	10.8	3.5	7.2 ± 2.1	6.4–8.0	< 0.01
ODI 4% (events/hour)	9.0	3.2	5.8 ± 2.1	5.0–6.6	< 0.01
Objective snoring > 40 dB (%)	39.0	20.7	18.3 ± 20.8	10.2–26.4	< 0.01
ESS (/24 points)	9.5	5.2	4.4 ± 4.3	2.7–6.0	< 0.01
PSQI (21 points)	6.6	4.8	1.8 ± 2.6	0.8–2.8	< 0.01

## Data Availability

All data can be will be made available on reasonable request.
